# Compatibility of Chitosan in Polymer Blends by Chemical Modification of Bio-based Polyesters

**DOI:** 10.3390/polym11121939

**Published:** 2019-11-25

**Authors:** Oscar Vernaez, Katharina Julia Neubert, Rodion Kopitzky, Stephan Kabasci

**Affiliations:** 1 Bio-based Plastic Department, Fraunhofer Institute for Environmental, Safety, and Energy Technology UMSICHT, Osterfelder Straße 3, 46047 Oberhausen, Germany; rodion.kopitzky@umsicht.fraunhofer.de (R.K.); stephan.kabasci@umsicht.fraunhofer.de (S.K.); 2 Fakultät für Angewandte Naturwissenschaften, Technology Arts Sciences Technische Hochschule Köln, Claudiusstr. 1, 50678 Cologne, Germany; k.j.wollek@gmail.com

**Keywords:** chitosan, grafting onto, bio-based polyesters, PHBV, maleic anhydride, glycidyl methacrylate

## Abstract

For some applications of bioplastics like food packaging or medical devices, applying additives can be necessary to avoid microbial activity and hinder biofilm or fouling formation. A currently promising additive is chitosan (CS), the deacetylated form of the biogenic scaffolding material chitin. Due to its hydrophilicity, chitosan is not compatible with most of the thermoplastic bio-based polymers like poly(lactic acid) (PLA) or polyhydroxyalkanoates (PHA). In this work, compatibilization between chitosan and two selected bio-based polyesters, PLA and poly(3-hydroxybutyrate-co-3-hydroxyvalerate) (PHBV), was enhanced by grafting maleic anhydride (MAH) and glycidyl methacrylate (GMA), respectively, onto polymer chains using peroxide. The success of grafting was confirmed via titration methods. The effects of grafting agent and peroxide concentrations on grafting reaction and the physical and thermal properties of the functionalized polyesters were investigated. Compounding of the functionalized polyesters with different weight portions of chitosan was accomplished in a discontinuous internal mixer by in-situ functionalization, followed by blending with chitosan. The titration method, scanning electron microscopy, DSC, FTIR and mechanical characterization of the composites showed good interfacial adhesion and suggest the formation of covalent bonds between functional groups of the polyesters and chitosan, especially for the samples functionalized with GMA. The molecular weights (Mw) of the samples showed a change in the molecular weight related to the thermal degradation of the sample. The Mw of the samples grafted with MAH are lower than those functionalized with GMA. Furthermore, integration of chitosan into non-functionalized PLA polymer matrix showed a nucleating effect, while for PHBV, the increase of crystallinity with the content of chitosan was only observed for grafted PHBV.

## 1. Introduction

Bio-based plastics or polymers are produced entirely or partially from natural resources. The majority of these materials are biodegradable in suitable composting plants or even in the environment within a few months to years. The most used and studied thermoplastic bio-based polymer is poly(lactic acid) (PLA). Under optimal conditions (60 °C, aerobic composting environment) PLA can degrade in 40 days [1]. Polyhydroxyalkanoates (PHA) are bio-based thermoplastic polymer synthesized by bacteria as energy storage materials that can be degraded under diverse environmental conditions (e.g., compost, anaerobic digestion, soil) and even in aquatic media. One potential application of bioplastics is for the packaging industry, where applying additives might be necessary to avoid microbial activity and hinder biofilm or fouling formation. Antimicrobial additives are typically solid fillers and are added to the thermoplastic matrix during the extrusion or compounding process.

A promising additive is chitosan, the deacetylated form of the biogenic chitin. On the one hand, this biopolymer offers a high availability in nature that can hardly be exhausted and on the other hand, it shows strong antimicrobial properties [2,3] due to its amino group based polycationic nature at the molecular level. Using chitosan as additive can increased the mechanical properties of the thermoplastic matrix and also give antimicrobial properties to the material [4].

PLA-chitosan composites show an inhomogeneous distribution of the chitosan, due to the incompatibility of both components. Chitosan exhibits a hydrophilic behavior due to its hydroxyl and amino groups and PLA and PHA are polyesters, which have a hydrophobic behavior [5].

The compatibility of two polymers in a blend can be enhanced through the modification of one or both components. One possibility is grafting co-monomers or functional groups to the polymer chains. Grafting is a method which is used to bond covalently small molecules, the grafting agents, onto the polymer chain.

This functionalization of polymers can be performed through radical initiation in solution or in the melt. The grafting mechanism and efficiency depend on the type of initiator, temperature and concentration of the grafting agent.

Maleic anhydride is suitable for functionalizing polyesters, not only because of the radical reactive double bond contained in the molecule, but also because of the reactive anhydride group towards nucleophiles. Maleic anhydride has a low potential for homopolymerization due to the steric inhibition of the 1,2-disubstituted double bond [6], although the formation of poly(maleic anhydride) under various condition was observed [7]. Regarding the modification of bio-based polyesters, the free radical grafting of MAH to PLA in the melt as well as in solution is known [4,8,9,10,11]. Grafted PHBV with MAH was synthesized by Avella et al. as a compatibilizer for improved binding of kenaf fibers (Hibiscus cannabinus) [12]. The effects of maleic anhydride grafting on polymer properties have been investigated more often for the polyhydroxyalkanoate PHB than for PHBV. For example, Chen et al. found that grafting onto highly crystalline PHB interfered with the regularity of the polymer chains and thus improved the morphological structure and hence the structural properties [13].

Due to the double bond available for radical polymerization reactions along with an epoxy group that can react with other chemical groups like carboxyl, hydroxyl anhydride or amines, GMA is another interesting monomer for functionalization reactions.

**Figure 1:**
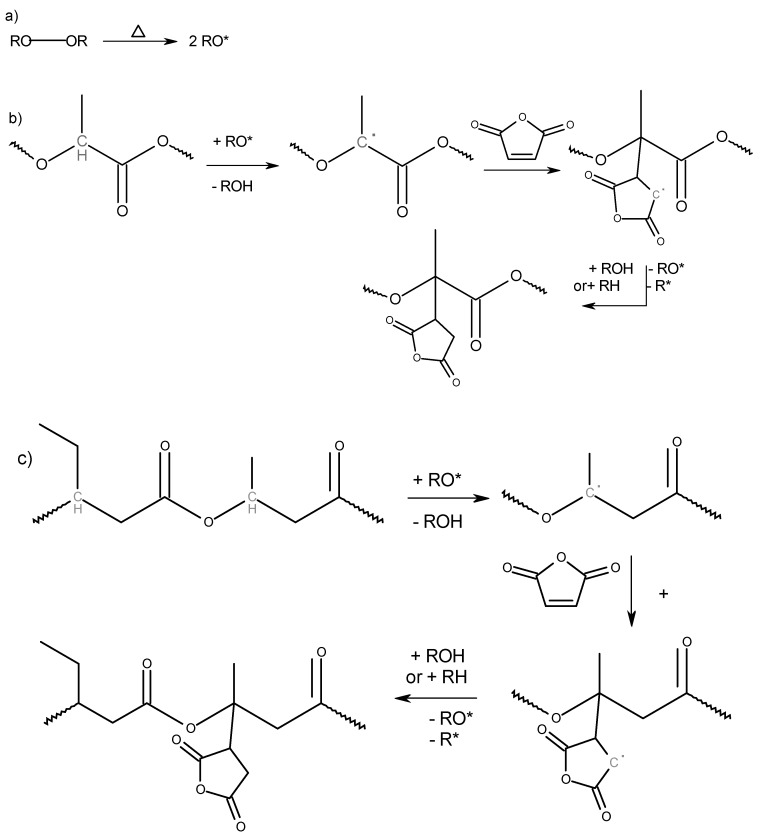
Reaction of maleic anhydride with poly(lactic acid) (PLA) and poly(3-hydroxybutyrate-co-3-hydroxyvalerate) (PHBV) using peroxides as the radical initiator. (**a**) Peroxide decomposition. (**b**) Grafting onto PLA. (**c**) Grafting onto PHBV.

**Figure 2:**
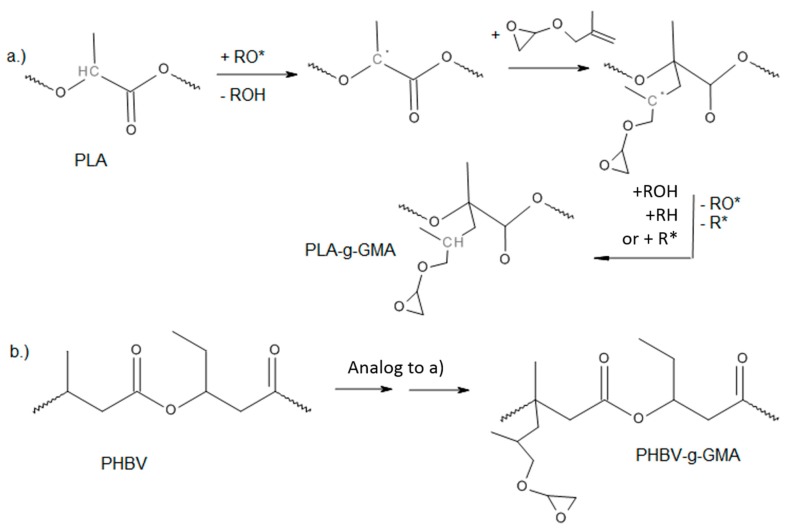
Reaction of GMA with PLA and PHBV using peroxide as the radical initiator. (**a**) Grafting onto PLA. (**b**) Grafting onto PHBV.

The general reaction mechanism of the free radical grafting using organic peroxide as an initiator is shown in Figure 1 and Figure 2. The first step is the homolytic thermal decomposition of the peroxide to form RO* radicals. Then, radicals are transferred to the polymer chain through the abstraction of a labile hydrogen to form a macroradical followed by the addition reaction of the macroradical to a double bond of the grafting agent. Finally, the chain reaction ends with the termination reaction when two radicals react with each other.

In the functionalization of PLA, the only possible site for hydrogen abstraction, which ensures good radical stabilization is the ternary-methyl-substituted carbon atom in the molecule. In PHBV, hydrogen abstraction at the ternary-ethyl-substituted carbon atoms would be conceivable.

Although the ethyl-substituted carbon atom would ensure a better stabilization of the free radical compared to the methyl-substituted carbon atom due to the higher positive inductive effect, Avella et al. considered that the insertion of MA mainly occurs onto hydroxybutyrate units, i.e., the methyl-substituted carbon atom, probably due to statistical, steric, and chemical effects [14]. Since the hydroxyvalerate content in the PHBV type used is only 3 mol%, it is assumed that, if grafting is successful, MAH will be most likely bonded to the methyl-substituted carbon atom of the butyrate moiety.

**Figure A1:**
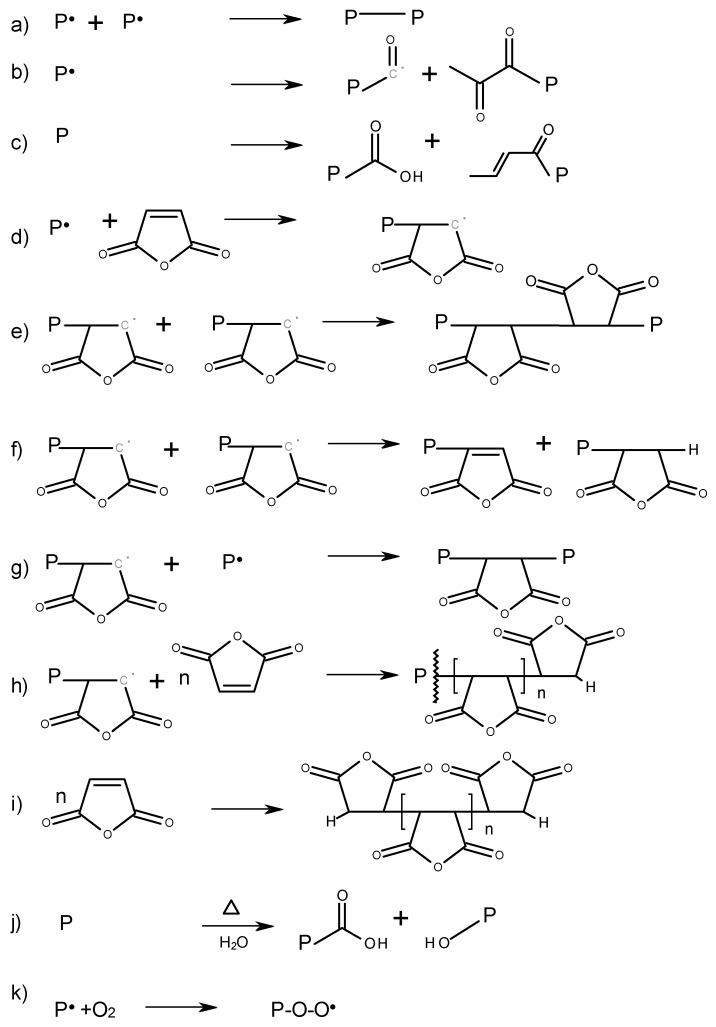
Possible side reactions for the MAH functionalization of a polyester. (**a**) termination reaction, (**b**) chain transfer, (**c**) thermal hydrolysis, (**d**) chain propagation, (**e**) termination, crosslinking, (**f**) termination, (**g**) termination crosslinking, (**h**) grafting with homopolymerization (**i**) homopolimerization, (**j**) thermal hydrolysis with water, (**k**) thermo-oxidation of radicals.

Along with the general grafting reaction, there are other side reactions that may occur. They include homopolymerization reactions of the MAH or GMA and the abstraction of other hydrogen atoms to form different radicals. Condensation reactions of MAH or epoxy groups with hydroxyl end groups that may be present in the polymer chains are also possible. Thermal and mechanical degradation of the polymer chains through the β-scission of PLA and PHBV are also possible [15]. Some of the side reactions for radical initiated functionalization of polymers with MAH are summarized in Figure A1, Appendix A. A termination reaction (a) leads to an increase in molecular weight, while (b), (c) and (i) lead to chain scissions. The products of reactions (d) to (g) are difficult to differentiate through analytic techniques. However, they all lead to a functionalized polymer. Reaction (h) refers to the homopolymerization of MAH. Similar side reactions might be expected from GMA monomers. In this study, the reaction kinetics and other reactions possibilities were not quantified.

Chitosan can react covalently with MAH groups through its hydroxyl groups or the amine groups. However, anhydrides show a higher reactivity to primary amines than to hydroxyl groups [16]. Therefore, it is assumed that the reaction between the primary amine groups of chitosan and the acid anhydrides is the predominant one.

It is known from the literature that the reaction of an acid anhydride with a primary amine, depending on the reaction conditions, can lead to the reaction products of a monoamide, a diamide or an imide [16,17,18]. Marechal et al. [19] have studied the influence of acid anhydrides, such as succinic anhydride, on polyamides and showed that they react with primary end amine groups to form imides. The reaction carried out in the melt took place in the presence of high temperatures. Li et al. revealed that an anhydride (4-chlorophthalate anhydride) reacts with methylamine to an imide compound in toluene under reflux [18]. The detection of imide groups can be demonstrated by a fourier-transformation infrared (FT-IR) spectroscopic examination [18,19].

The incorporation of such reactive groups like MAH and GMA in the thermoplastic matrix should enhance the compatibility with chitosan due to the reaction of anhydride and epoxy groups with the primary amine and hydroxyl groups of the chitosan. A better distribution of chitosan particles as well as an improvement of mechanical properties are expected.

In this work, the compatibility of PLA and poly(3-hydroxybutyrate-co-3-hydroxyvalerate) (PHBV) with Chitosan is enhanced through the functionalization of the thermoplastic polyesters with maleic anhydride (MAH) and glycidyl methacrylate (GMA) via reactive blending in an internal mixer.

## 2. Materials and Methods

### 2.1. Materials

Chitosan 90/200/A1 (90% degree of deacetylation, viscosity 170 cps) was supplied by BioLog Heppe GmbH (Queis, Germany). 2,5-Dimethyl-2,5-di-(tert-butylperoxy)-hexan (DTBP) with an activation energy Ea = 155.5 kJ/mol and an A-parameter of 1.68 × 10^16^ from Pergan GmbH (Bocholt, Germany) was used as initiator. Maleic anhydride and glycidyl methacrylate from Sigma-Aldrich (Taufkirchen, Germany) were used as grafting agents. Poly(3-hydroxybutyrate-co-3-hydroxyvalerate) (Enmat Y 1000P with 3 Mol% valeric acid content) from TianAn Biological Materials (Ningbo city, China) and PLA 2003D from Nature Works (Blair, NE, USA) were used as biopolymer matrices.

### 2.2. PLA and PHBV Grafting in the Melt

For functionalization in the melt, an internal mixer Lab-Station 350E (Brabender GmbH & Co. KG, Duisburg, Germany) was used. The mixer was equipped with continuous injection of nitrogen to decrease thermo-oxidative reactions with oxygen. The temperature was set to 180 °C with a rotation speed of 60 rpm. The previously dried (12 h at 130 °C) polymer pellets were fed into the mixing chamber after it had reached the set-point temperature. Torque and temperature were recorded once the polymer was fully added. Exactly 4 min after the addition of the polymer, DTBP was added together with the grafting agent (MAH or GMA). Two different peroxide concentrations (0.3% and 0.6% ^w^/_w_, representing 1.03 × 10^−5^ and 2.07 × 10^−5^ mol/g respectively) and two different concentrations of grafting agent (3.0% and 10.0% ^w^/_w_) were studied for each polymer. Mixing proceeded for 7 min after the addition of the additives. Table 1 shows the samples prepared for grafting.

Purification was performed by dissolving the product from the internal mixer in dichloromethane, filtrating the solution using a paper filter MN 615 (4 to 12 μm average retention capacity) followed by precipitation in ethanol.

### 2.3. Chitosan Composites Preparation

PLA and PHBV with the highest concentration of grafting agent (10% *w*/*w*) were compounded with chitosan in the same internal mixer. The concentrations of peroxide used were selected after estimating the grafting grades. A concentration of wt. 0.6% resulted for the PLA samples and 0.3% for the PHBV samples. The blends were compounded according to the procedure described in the previous section, with the addition of DTBP and the grafting agent after 4 min and the chitosan powder was added to the internal mixer after 8 min. The final mixtures were compounded for five more minutes before they were taken out of the mixer and cooled at room temperature. The processing parameters used were also 180 °C and 60 rpm. The chitosan concentration was chosen to obtain two different molar ratios between the amino groups from chitosan and the degree of grafting, i.e., 10:1 and 1:1. That means that the chitosan concentration in the blend depends on the degree of grafting obtained for each polymer. Table 2 shows the composites prepared with chitosan.

### 2.4. Analytical Methods

The degree of grafting, in mass percent in relation to the polymer mass or as a percentage of successfully covalently bonded grafting agent, was determined through titration for PLA and PHBV grafted with MAH and GMA.

The titration methods for determining the grafting degree of MAH and GMA differ from each other. The error of the methods was determined using two exemplary samples with the highest grafting degree.

#### 2.4.1. Determination of the Degree of Grafting of MAH

A titration method was used to determine the degree of successful grafting of MAH on the polymer chain. The basic principle of the analysis method is the hydrolytic cleavage of the covalently bonded anhydride groups into dicarboxylic acids for their subsequent neutralisation with a base (potassium hydroxide solution).

Following Hwang et al. [9], a solvent mixture of chloroform, methanol and fully demineralised water (3:2:0.2 *v*/*v*/*v*) was prepared. The addition of water for the hydrolysis of the acid anhydrides and the ratio in relation to the total solvent mixture were taken from the standard DIN EN ISO 2114:2000. The samples were dissolved at 60 °C with phenolphthalein. By titration with a solution of potassium hydroxide, the acid value (AV) was measured.

The degree of grafting of maleic anhydride *MAH*% was determined from the acid value using the following equation:

(1) $$MAH\%=\;\frac{AV\cdot M_{MAH}\cdot 100\%}{56.1\cdot \;10^{3}}=\;\frac{AV\cdot M_{MAH}}{561},\;\;\left[\%\;w/w\right]$$

where *M_MAH_* is the molecular weight of the maleic anhydride. The average standard deviation was 0.05%.

#### 2.4.2. Determination of the Degree of Grafting of GMA

The degree of grafting of GMA on the polymer was measured according to the standard DIN EN ISO 3001, where the determination of the epoxy equivalent is described. From this value, the mass of polymer per epoxy functional group is obtained. The background of this analytical method is the in-situ production of hydrogen bromide by the addition of a bromide containing agent and the subsequent titration with perchloric acid. HBr breaks up existing epoxy groups through a nucleophilic attack. At a transition point of about pH 1, it can be assumed that all epoxy groups reacted and hydrogen bromide was free in solution.

The calculation of the epoxy equivalent (*EE*) can be obtain from Equation (2):

(2) $$EE=\;\frac{1000\cdot m}{\left(V_{1}-V_{0}\right)\cdot \left(1-\frac{T-T_{s}}{1000}\right)\cdot c},\;\;\left[\frac{g_{sample}}{mol_{Epoxid}}\right]$$

where *EE* is the epoxy equivalent expressed in grams of sample per mol epoxy group, *m* is the mass of the sample in g, *V_0_* is the volume of perchloric acid needed for the titration of a blank with no epoxy groups, while *V_1_* is the volume of perchloric acid used for the titration of the sample. *T* is the temperature in °C of the perchloric acid solution during the titration of the blank, while *T_s_* is the temperature of the perchloric acid solution during the titration of the sample. The concentration of the perchloric acid solution is *c* in Equation (2).

Using the epoxy equivalent and taking into account the molar mass of glycidyl methacrylate, the grafting degree of GMA (*GMA%*) was calculated in % *w*/*w*, as presented in Equation (3),

(3) $$GMA\%=\;\frac{M_{GMA}}{EE}\cdot 100\;\%,\;\;\left[\%\;w/w\right]$$

where *M_GMA_* is the molecular weight of the GMA. The average standard deviation was 0.34%.

#### 2.4.3. Determination of Grafting Yield

The grafting yield *Y* is the percentage of the grafting agent that successfully attached to the polymer matrix and is calculated from the degree of grafting from equation 1 or 3 in relation to the initially added mass of MAH (*m_MAH_*) or GMA (*m_GMA_*) according to Equation (4):

(4) $$Y=\;\frac{MAH\%\;or\;GMA\%\cdot m_{Polymer}}{m_{MAH}\;or\;m_{GMA}},\;\;\left[\%\right]$$

### 2.5. Instrumental Methods

Fourier transform infrared spectroscopy (FT-IR) was made on a Tensor 27 instrument equipped with a Platinum Diamond ATR unit (both: Bruker Optik GmbH, Esslingen, Germany) The samples were measured with a resolution of 4 cm^−^^1^ in the wavelength range from 4000 to 400 cm^−^^1^. Sixteen scans were accumulated.

Scanning electron microscopy (SEM) was performed in a TESCAN Vega3 equipment with 20 kV (TESCAN Brno, Czech Republic). Samples for SEM were prepared from broken hot-pressed plates with sputtered gold coating.

Differential scanning calorimetry (DSC) measurements were run in a DSC 204 F1 Phoenix apparatus from Netzsch (Selb, Germany). The samples were heated to 220 °C to erase crystalline memory and then cooled to 0 and −30 °C for PLA and PHBV, respectively at 10 °C/min. A second heating at 10 °C/min was performed up to 240 °C for PLA and to 220 °C for PHBV. To calculate the crystallinity of the polymers, the reference enthalpy for100% crystalline polymer (Δ*H_m_^0^*) used for PLA was 93.6 J/g [20] and 146 J/g for PHBV [21].

Gel permeation chromatography (GPC) measurements were performed in a SECcurity GPC/SEC System instrument with three columns (PSS GmbH, Mainz, Germany) to cover the range from 100 to 10^6^ Da, using 1,1,1,3,3,3-hexafluor-2-propanol (HFIP) as eluent and PMMA as reference. Additionally, multi-angle light scattering (MALS) from PSS coupled with the GPC system was used to determine the absolute values of the molecular weight of PLA and PHBV samples. Blends with chitosan were not evaluated with GPC due to the insolubility of the chitosan.

### 2.6. Tensile Testing

Tensile tests were executed in a universal tensile test machine Instron 5567A (Instron GmbH, Darmstadt, Germany) with a maximal charge of 5 kN. Type 2 samples for the tensile tests were cut from 2 mm-thick hot pressed plates using a programmable milling cutter and the tests were conducted according to the international standard DIN ISO 527 with at least five replicates for each sample.

## 3. Results and Discussion

### 3.1. Grafting

The grafting method used in this work is based on a reaction initiated by radicals. The kinetics for peroxide decomposition is well known and has an Arrhenius dependence with temperature and concentration.

In this case, DTBP was used as the initiator at 180 °C, with a half-life time of 35 s.

#### 3.1.1. Functionalization in the Internal Mixer

**Figure 3:**
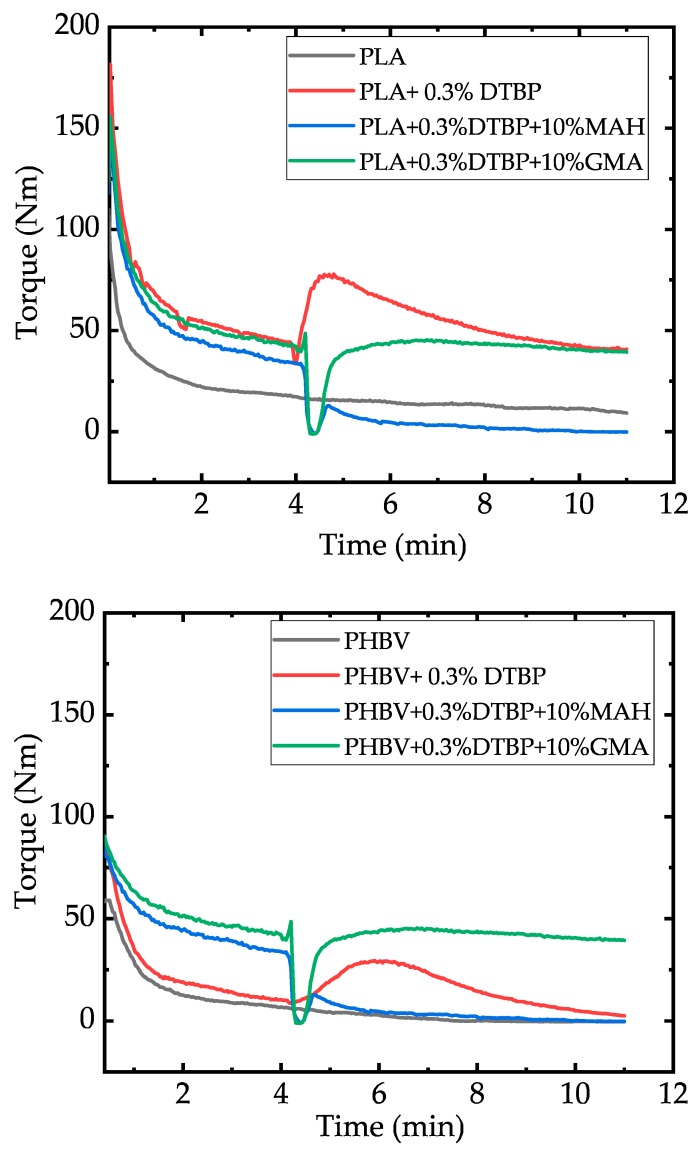
Plastograms of PLA (**upper**) and PHBV (**lower**) in the internal mixer.

Plastograms (Figure 3) were recorded to compare the behavior of the different materials. Standard runs were performed with the neat polymer (PLA and PHBV) without any additives. A second reference contained the base polymer and peroxide but no grafting agents. Finally, the trials with peroxide and with MAH or GMA were conducted.

The starting time (minute 0) is considered when the polymer pellets were added in the internal mixer after being running for about 2 min. In all the trials, the torque decreases progressively due to the melting of the polymers. After four minutes, the peroxide, i.e., the radical initiator, was added to the internal mixer. The increase in the torque for the trials with peroxide but without addition of MAH or GMA is due to the radical reaction, leading to an increase in molecular weight with an increase in the viscosity because of chain entanglements. The steep increase of the torque corresponds to the quick decomposition of DTBP with a half-life time of merely 35 s at 180 °C.

Peroxide crosslinking with organic peroxides have been presented previously as being effective in crosslinking and the increase of melt strength of PLA [22,23]. Parallel to the chain extension reaction, some chain scission occurs as a result of the high temperatures and shear rates affected by the constant speed mixing [24]. The resulting thermal degradation can be observed as a decrease in torque after the increase due to the extension reaction. The same observation can be made for the PHBV polymer, so it can be inferred that the chain extension reaction as well as the thermo-mechanical degradation in time occur in this polymer in the same way as in PLA. Nevertheless, if MAH is added to the mixer, the increase in viscosity is no longer seen. This can be regarded as evidence of the consumption of radicals by the MAH grafting agents. When GMA is added to the internal mixer, a first drop in the torque is seen due to the low viscosity of the added additive; then, the viscosity begins to raise to a small extend, suggesting the occurrence of some branching.

The success of the grafting reaction of MAH and GMA was evaluated through titration. First, non-purified products that came out from the internal mixer were measured. After these products were purified to separate possible low molecular weight residues (unreacted grafting agents, oligomers formed from the grafting agents etc.), the polymers were titrated again to estimate the degree of covalent grafting after purification.

For purification, the blend is dissolved in dichloromethane, filtrated and then precipitated in ethanol. The samples treated with MAH and collected from the internal mixer showed a yellow to brown color, while after purification, the samples were white. Detyothin et al. have reported the same coloration behavior and they attributed it to the undesirable side reaction of free radical grafting. Polymaleic acid or MAH homopolymers have the same coloration, thus, the coloration can be assumed as evidence of the polymerization reactions of the grafting agents. Homopolymers of MAH, as well as unreacted grafting agents were removed by the purification step. In the case of PLA-g-GMA, no change in color was observe after purification.

**Figure 4:**
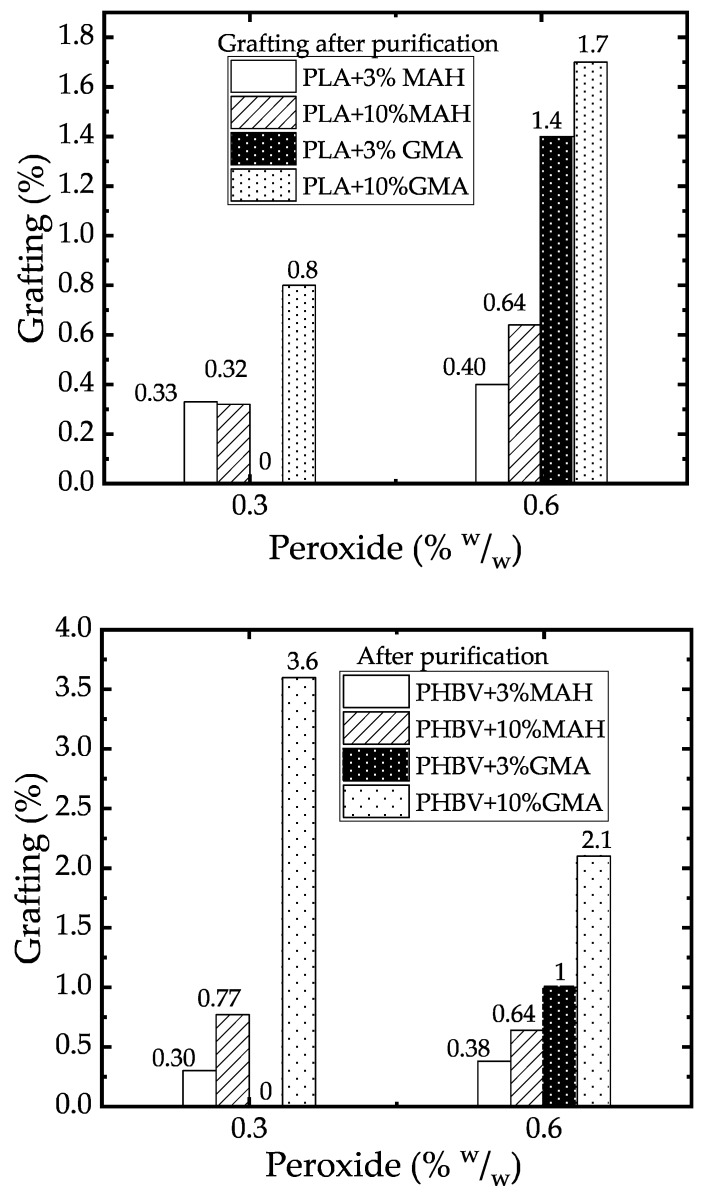
Grafting in internal mixer after purification, solution and precipitation (**top**: PLA, **bottom**: PHBV).

From Figure 4, looking at the values after purification, it can be inferred that PHBV functionalization was more effective than PLA grafting. This difference was not detectable from the plastograms recorded in the internal mixer. The unpurified samples of PLA and PHBV show similar functionalization. After purification, however, PHBV shows higher grafting grades than PLA under the same reaction conditions. This may be due to the higher expected mobility of the PHBV molecules, which allows the monomer diffusion. The absolute values of torque indicate that the viscosity of the PHBV melt is significantly lower at the process temperature than of the PLA. Hence, peroxide and grafting agent diffusion in the thermoplastic matrix are higher and more efficient grafting reactions are expected. For PHBV, a higher functionalization was obtained for a peroxide concentration of 0.3% than for 0.6%. This indicates an increase of homopolymerization or termination reactions due to an excess of radicals.

For the combinations of low concentration of peroxide (0.3%) and low concentration (3%) of grafting agent GMA, there is no covalently bonded functionalization detectable after the purification procedure.

To avoid errors from the polymer end groups, the apparent grafting grades for samples without MAH or GMA were measured. The titration method for MAH was performed using the polymers after processing in the internal mixer at 180 °C with and without peroxide addition. It was determined that the concentration of acid groups for PLA was 6.03 × 10^−6^ mol/g without and 2.61 × 10^−5^ mol/g with 0.3% of DCP, while for PHBV 5.93 × 10^−5^ mol/g were present without and 1.96 × 10^−5^ mol/g with peroxide. Since the raw PLA is end-capped, the concentration of acid groups on the unprocessed polymer could not be determined. It is assumed that the acid groups measured on the processed PLA were produced by hydrolysis and thermal degradation [24]. The grafting of MAH on PLA and PHBV was corrected with the concentration of acid groups found for PLA and PHBV processed without any monomer. It is then assumed that the same amount of acid groups was formed due to hydrolysis.

#### 3.1.2. Molecular Weight of Functionalized PLA and PHBV in the Internal Mixer

**Figure 5:**
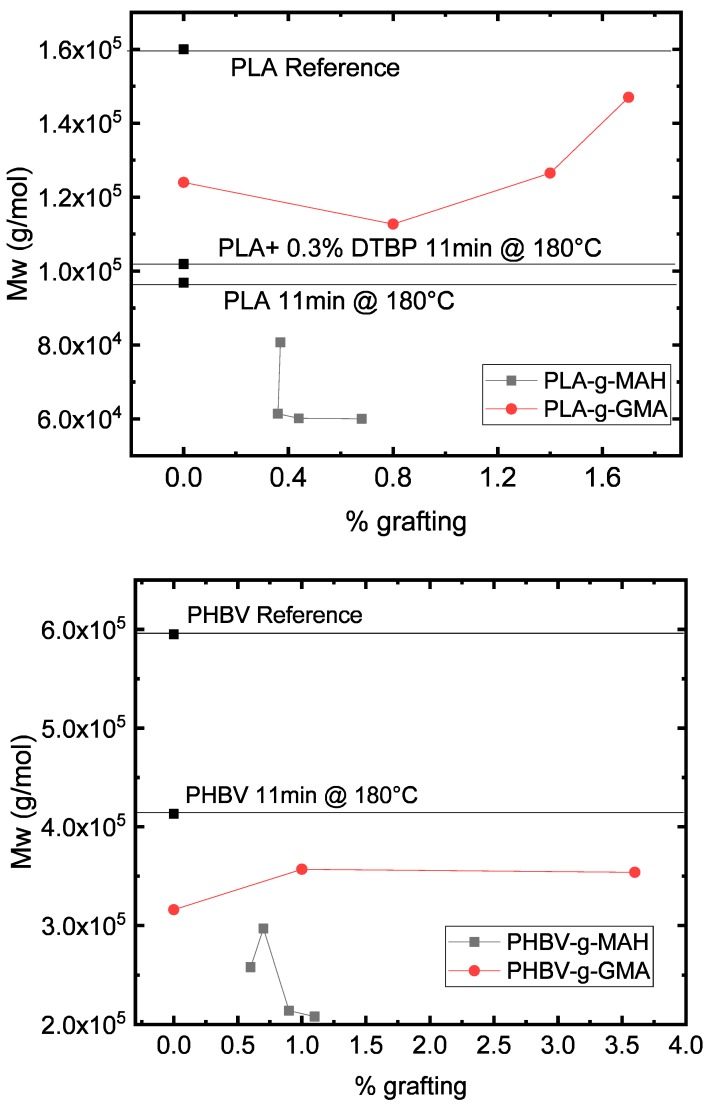
Molecular weight of the grafted PLA (**upper**) and PHBV (**lower**).

Figure 5 shows the molecular weights of the neat polymers as references, the neat polymers after 11 min residence time in the internal mixer at 180 °C, the polymers after this treatment and the addition of peroxide after 4 min, and the polymers after grafting and purification, measured by GPC. The thermal degradation of PLA in the internal mixer is around 40% (reduction of Mw from 1.59 × 10^5^ g/mol to 0.96 × 10^5^ g/mol). The reaction with peroxide does not increase significantly the molar mass of this polymer. For PHBV, the thermal degradation is about 30% (reduction of Mw from 5.95 × 10^5^ g/mol to 4.13 × 10^5^ g/mol) due to the mixing and temperature. Nevertheless, the addition of peroxide leads to a partially non-soluble product so that no reliable GPC-measurement can be performed. Looking at the plastograms in Figure 3, the reaction of PHBV with peroxide did not lead to an exceedingly high torque result after 11 min of reaction. The reason for the formation of non-dissolvable polymers in the reaction of PHBV with DTBP could not be identified and needs further investigation.

Observing the molecular weight of the samples functionalized with MAH, a significant reduction of the molecular weight can be seen in both polymers. This is in accordance with the reduction of viscosity observed in the plastograms. Thus, MAH promotes the degradation of the polymer and reduces the molecular weight to values even lower than the reference after 11 min in the mixer at 180 °C. This phenomenon was observed by Raquez et al. [6]. The authors described that independent of the processing temperature, the excess of radical leads to a competition of chain branching by termination reaction and *β*-scission. They assumed a hydrogen abstraction in the tertiary carbon of the functional group and further *β*-scission of the ether group in the backbone to form a double bond with terminal MAH and a RO* macroradical. A similar behavior was observed by Kim et al. [25] while functionalizing polycaprolactone (PCL) with MAH. They observed chain scission with increasing temperature. However, they did not discuss a possible mechanism.

On the other hand, the polymers functionalized with GMA show a higher molecular weight than those grafted with MAH. For PLA (Figure 5, upper), the molecular weight was even higher than the reference treated only with peroxide. Hence, possible reactions of the epoxy groups with the products of chain cleavage of PLA may occur, compensating the loss of molecular weight. Kim et al. [25] also observed the same difference in changes of molecular weight while functionalizing PCL with both MAH and GMA. For GMA functionalization, they attributed the increase in molecular weight and broadening of the distribution to recombination and termination reactions of the radical after the functionalization. No evidence of *β*-scission was observed. These reactions do not seem to take place in PHBV (Figure 5 lower). A concrete mechanism cannot be deduced from these results, however, it is important to notice the difference in the reaction of both polymers due to the different stability of the radical after the hydrogen abstraction. The results presented in the literature showed ambiguous trends. Domenichelly et al. [11] found an increase of the molecular weight with increasing the MAH/Peroxide ratio and on the other hand, Detyothin et al. [10] found a decrease in Mw with the increase in the %MAH grafting agent.

### 3.2. Chitosan Composite

#### 3.2.1. Compounding

Once the results of the grafting grade of each sample were obtained, the corresponding chitosan concentration for the compounds was selected. Considering a degree of deacetylation of 90%, the molar concentration of amine groups in relation with the functional groups of the grafted polymers (MAH or GMA) were calculated to have either a 1:1 or a 1:10, i.e., n_MAH_:n_NH2_ = 1 or n_MAH_:n_NH2_ = 10. The calculations were done assuming that the grafting reaction occurs to the same extent as that observed in the previous section without chitosan.

Even though the presence of chitosan might influence the reaction progress, no radical reaction is expected to take place, neither with the glucosamine nor with the amine.

**Figure 6:**
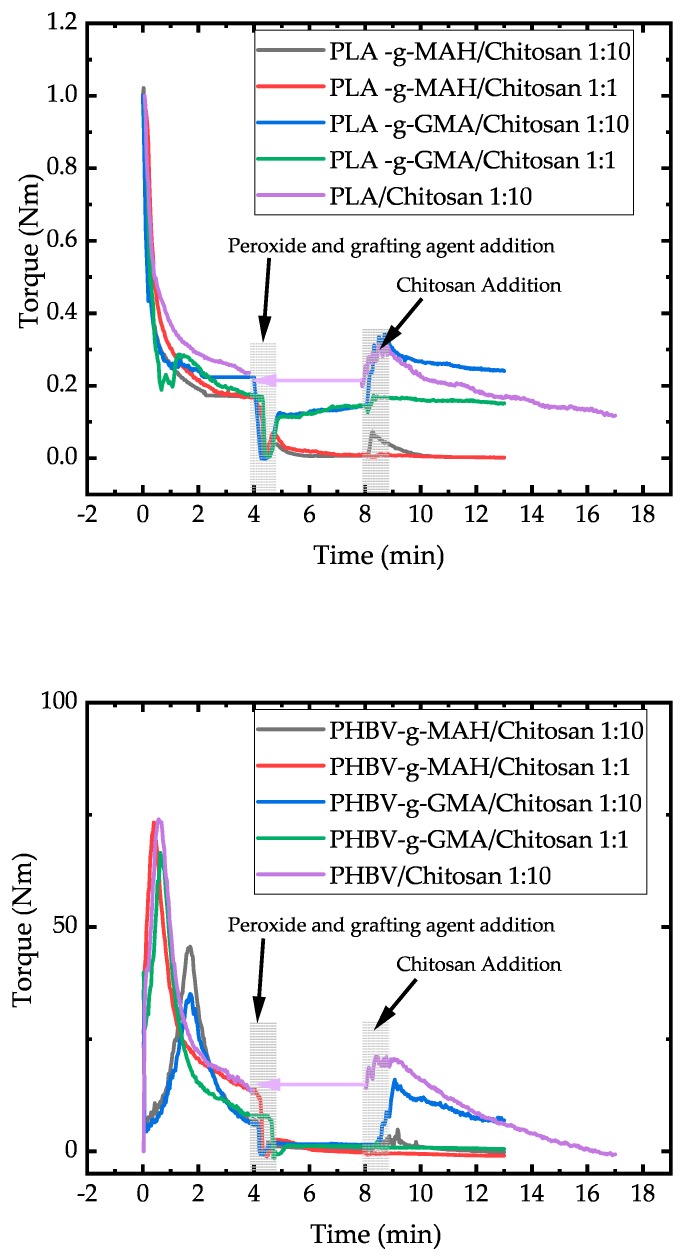
Plastograms of compounding grafted PLA (**up**) and grafted PHBV (**down**) with chitosan. By reference PLA/Chitosan 1:10 and PHBV/Chitosan 1:10, the addition of Chitosan was done at 4 min, but it was shifted to 8 min for comparison.

In Figure 6, the plastograms of the corresponding compounding are presented. A similar course of the curves as in the plastograms in Figure 3 can be seen for the polymer melting and the addition of peroxide and grafting agents after 4 min. At 8 min, the addition of chitosan powder to the internal mixer increased the viscosity of all of the samples, because the chitosan does not melt and behaves as a solid filler. Furthermore, the viscosity continues to decrease due to the thermal degradation of the polymer matrix, as expected from Figure 3. However, final torques are higher than those obtained after mixing the polymer for 13 min without any additive.

#### 3.2.2. Reaction Mechanism

**Figure 7:**
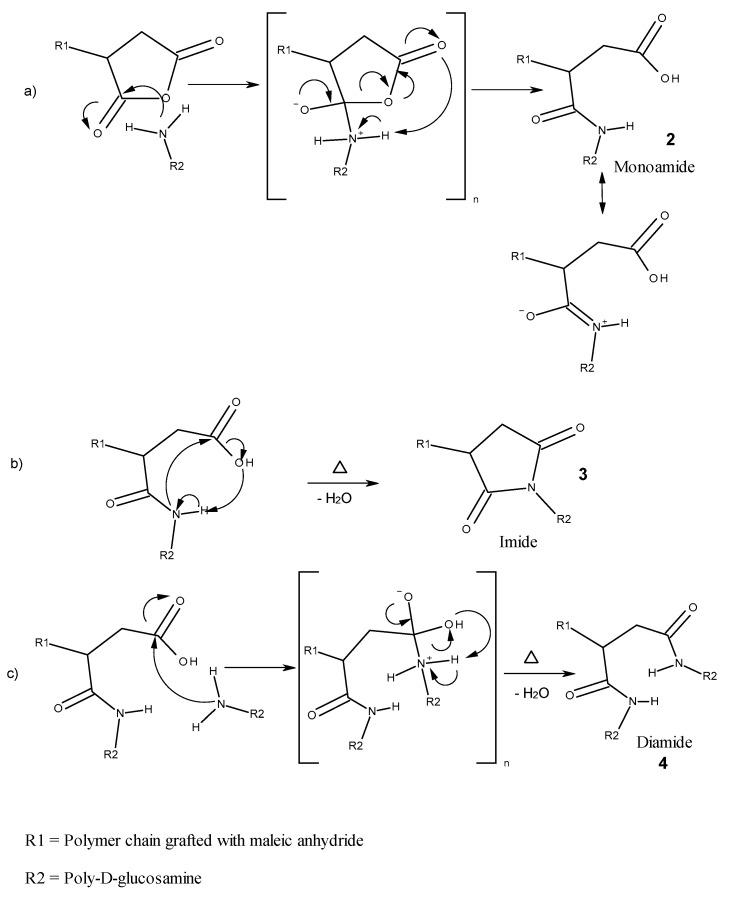
Possible reactions of the amine groups of chitosan with maleic anhydride (MAH) grafted polymer.

The possible reaction mechanisms and products for the reaction of primary amines with substituted maleic anhydride are presented below (see Figure 7). In general, the amine acts as a nucleophile, while the acid anhydride acts as an electrophile. In a first step (a), the nucleophilic nitrogen atom of the amine group with its free electron pair attacks the carbonyl carbon atom of the anhydride forming a tetrahedral intermediate (**1**). After rearrangement of the bonds, the anhydride breaks up into a resonance-stabilized monoamide (**2**). Because the free electron pair of the secondary amine participates in the resonance stabilization, it is less nucleophilic than the primary amine, but some high-energy input can lead to a nucleophilic attack on the carbonyl carbon of the acid group (b). When water is split off, an imide (**3**) is formed. Starting from the monoamide, an excess of amine groups can lead to another nucleophilic attack on the remaining carbonyl carbon, resulting in the salt form of the diamide (c). The reaction to the diamide (**4**) takes place after energetic activation and the elimination of one mole of water.

The primary hydroxyl group on the 6-carbon atom of glucosamine can also react with an acid anhydride. In this case, the nucleophilic attack occurs through the oxygen atom of the hydroxyl group to a carbonyl carbon of the acid anhydride. Through rearrangements of bonds and elimination of one mole of water, an ester forms. Furthermore, condensation reactions between carboxyl end groups of the polyesters used with hydroxyl groups of polyglucosamine or polymer end groups are possible, but these are the least likely due to low concentrations.

If the polyester is glycidyl methacrylate grafted, the epoxy functionalization is decisive for the covalent bonding with chitosan. Epoxides react with nucleophiles in a ring opening reaction. Due to the tension of the epoxide ring, the carbon atoms in an epoxy ring are reactive electrophiles. In asymmetric epoxides, i.e., those with different substituents at both ends, stereoselective reactions take place. On which side the nucleophile attacks depends on various factors. A distinction can be made between an SN2 mechanism, in which only a nucleophilic attack on the less substituted carbon atom occurs and an unstable intermediate stage develops after the leaving group was eliminated. Regarding chitosan, two possible nucleophiles have to be considered: the primary amine and the primary hydroxyl group. Due to the slightly acid character of chitosan (pKa = 6.5) and the strong nucleophilicity of amines, the ring-opening reaction may follow the SN2 mechanism.

**Figure 8:**
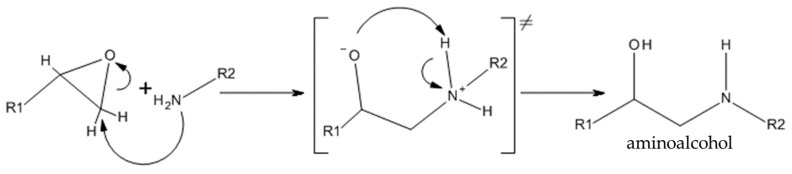
Possible reaction of the amine group of chitosan with the epoxy group of the GMA functionalized polymer.

If the nucleophilic attack occurs through the primary amine, the sterically unhindered carbon atom of the epoxy ring is attacked. The ring opening leads to a transition state, with the oxygen atom carrying a negative charge and the nitrogen atom a positive charge. The oxygen atom abstracts a hydrogen atom from the nitrogen atom, thus forming a secondary amine, more precisely, an amino alcohol (see Figure 8). The reaction products formed in the described reaction contain hydroxyl groups, which act as proton donors and thus contribute to an autocatalytic reaction.

If the nucleophilic attack is based on a hydoxyl group on the chitosan, an acid catalyzed reaction is assumed. It has been described in the literature that the reaction between epoxides and secondary hydroxyl groups can be accelerated by using a high concentration of imidazole [26]. It was confirmed that an excess of epoxy groups and imidazole leads to a stronger branching than with a stoichiometric ratio of epoxy to hydroxyl groups [26].

Since the reaction of MAH and GMA groups with the amine groups of the chitosan are well known and since it is possible to determine the amount of MAH in the composite through titration, the chemical reaction between functional groups on the grafted polymers can thus be followed by quantifying the amount of functional groups after compounding. The more functionally the groups have reacted, the higher the chemical compatibility between the two phases.

#### 3.2.3. Reactions after Compounding

The number of amine groups that eacted with the grafted polymer were calculated through titration of the final compounds. Then, the concentration of MAH obtained was compared with the expected concentration from previous results. For this titration, no purification was performed, because of the non-solubility of the chitosan in the solvents used. The comparison was then made against the grafting grade of the unpurified samples. If the amine groups have reacted with all MAH available, the MAH concentration measured after compounding with chitosan should be zero, otherwise, a positive concentration of MAH means that some MAH groups or carboxyl groups from chain cleavage did not react with amine as expected. Nevertheless, is has to be assumed that a non-countable amount of MAH, that has reacted with the chitosan, may not have been grafted onto the polymer previously. Titration of the GMA grafted samples was not possible. The presence of chitosan did not allow a proper titration of the epoxy groups. Table 3 shows the results of the titration of polymers grafted with MAH and compounded with chitosan.

The percentage of MAH, which has reacted with the amine groups, was estimated from the titration results of MAH content. More than 90% of the available MAH reacted with the amine groups, independently of the grafted polymer or of the mole relation of amine groups. That was not expected for a n_NH2_:n_MAH_ = 1 relation, taking into account that only amine groups on the interfacial region between chitosan and the thermoplastic matrix, were available for reaction. One possible explanation is the migration of unreacted MAH into the chitosan matrix. It is also possible that the carboxylic acid groups produced via chain scission of PLA or PHBV were reduced due to the incorporation of chitosan during the mixing. Based on the percentage of reacted MAH it can be inferred that most of the functional groups grafted to the polymer can react with the chitosan’s amine groups to enhance the chemical compatibility of the composite.

#### 3.2.4. Thermal Properties of the Grafted Polymers and Chitosan Blends

Table 4 presents the results of the thermal properties measured with DSC. In case of the materials without chitosan, purified samples were analyzed.

It is well known that *Tg* is an important indicator for the miscibility of components in a blend. In a completely miscible blend of two polymers, only one *Tg* is expected with a value between the *Tg* values of the pure polymers. If two components are only partially miscible, the *Tg* value of each component phase will slightly be affected by the other one. The degree of this effect is usually composition-dependent.

The glass transition temperature *Tg* of the functionalized PLA, processed at 180 °C, was 62.1 °C and the *Tg* of the PHBV was 6.2 °C. Grafting of MAH on PLA leads to a decrease in *Tg*. The same effect has been reported in [9]. A plasticizing effect of unbound MAH is supposed to be responsible for this observation, assuming no effect of the reduction of the molecular weight on the glass transition. On the other hand, no significant trend was found for the materials grafted with GMA in this study. This is in opposition to the decrease of the glass transition temperatures previously reported in the literature [27,28]. Regarding PHBV, the functionalization with MAH leads to slightly higher *Tg* values, while the grafting with GMA effects a significant reduction of the *Tg*. This is probably due to the smaller size of the functional group.

The glass transition of chitosan may vary depending on water content, molecular weight and deacetylation grade and its value is controversial. However, since chitosan is a polysaccharide it can be assumed to be higher that its decomposition temperature. Using standard DSC methods with linear temperature ramps, like in this case, the glass temperature of chitosan cannot be determined. The *Tg* of neat PLA and PHBV composites with chitosan decreases in relation with the neat polymers. For grafted PLA, though, the *Tg* was higher for the composites. Composites of grafted PHBV with chitosan show an increase in *Tg*, as was noticed for the grafted PHBV without chitosan. This shift in *Tg* indicates an enhanced compatibility of the blend partners, although the magnitude of the shift is too small to draw a firm conclusion.

The PLA used in this study crystallizes very slowly, as can be confirmed by the DSC of the raw material. However, a non-expected crystallinity was observed for the samples of PLA after adding peroxide. Usually, the crystallinity of such a sample would be expected to decrease due to the interruption of crystalline sequences by the branching introduced by the addition reactions. Because of this unusual behavior, the measurement was repeated for the same sample with a similar result. Additionally, an increase of crystallinity was observed for samples PLA_3G1P and PLA_3G2P. A more detailed study would be necessary to validate these results. For the following discussion of this work, the crystallization values of these samples are not considered.

**Figure A2:**
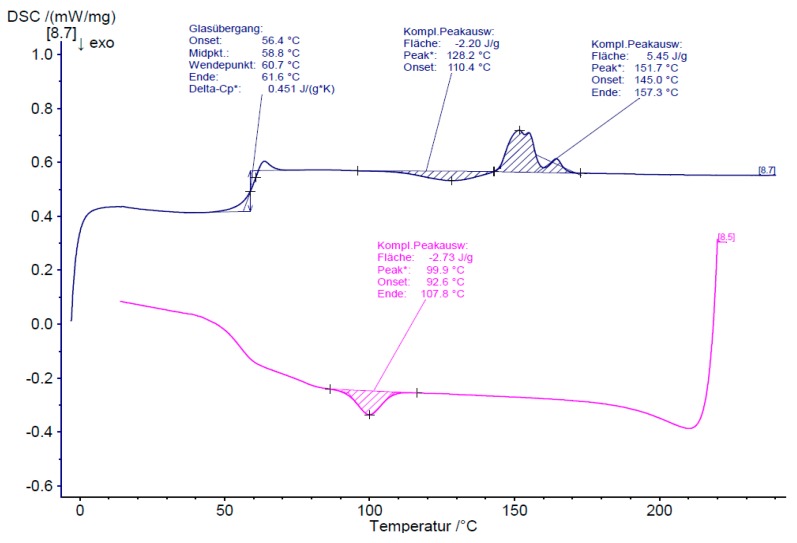
Dynamic scanning calorimetry of the reference PLA/Chitosan composites.

**Figure A3:**
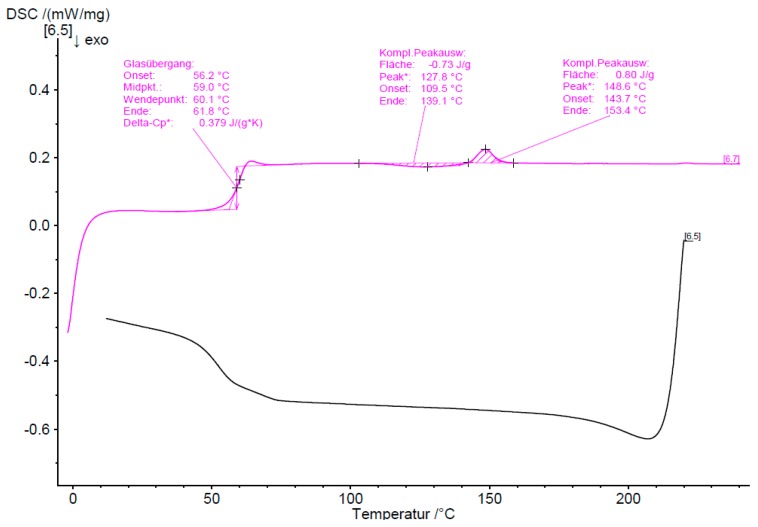
DSC of PLA-g-GMA/Chitosan 10% composite.

**Figure A4:**
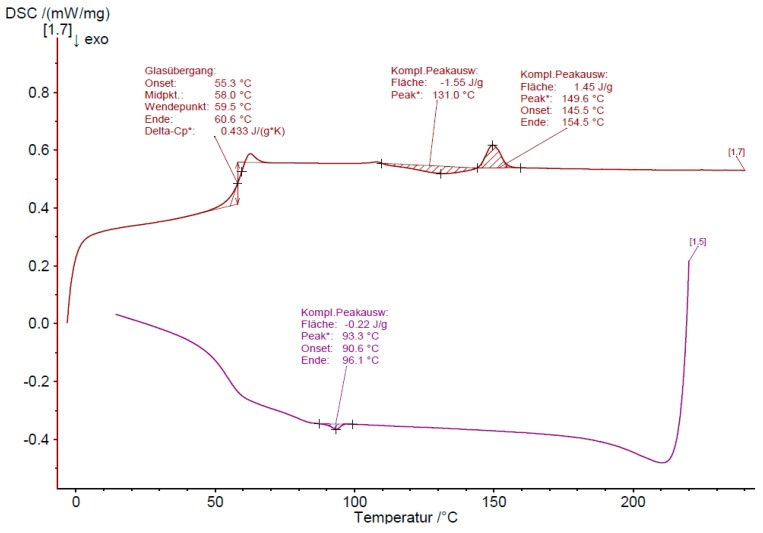
DSC of PLA-g-MAH/Chitosan 10% composite.

The addition of chitosan to neat PLA increases the crystallinity to 4%, which may be an indicator of a nucleating action of chitosan. Another striking feature of this sample is the splitting of the melt peak into three smaller peaks (Figure A2). Such split peaks are not observed in the functionalized polymers compounded with chitosan (Figure A3 and Figure A4). The occurrence of the shoulder peaks can be explained either by melting of three different crystal types or by defects of existing crystallites [29]. The PLA chains might be arranged around the chitosan particles in uniformly folded crystals. This finding is consistent with other authors who found that chitosan in PLA composites promotes crystallization and acts as a nucleating agent [30]. However, in the publication of Gupta et al. [31], in which the effect of functionalized chitosan on the crystal formation of PLA was investigated, chitosan was entitled ‘pseudo-nucleating agent’. This statement is justified by the assumption that chitosan acted more as a promoter for the interaction of PLLA with PDLA and thus ensured uniform and faster nucleation due to the formation of stereo complexes. This may not apply to the samples used in this work because the ratio of sterocomplex in our sample is unknown. In contrast, Fortunati et al. [32] found that the addition of chitosan to the PLA did not affected either the melting temperature nor the crystallinity of the blend.

The melting temperatures observed in the PLA studied here were not affected by the introduction of chitosan to the blend as Fortunati et al. found. This may be an indicator of a bad compatibility between chitosan and PLA. There is also no evidence of nucleation in the grafted PLA samples. The reason for this could be that the grafted monomers and the homopolymers present act as disruptive factors for a parallel arrangement of the polymer chains in folding crystals.

The melting temperatures of the functionalized PHBV-chitosan composites are lowered by chitosan, which could indicate a binding of chitosan to the polymer chains. The determined crystallinity degrees of the PHBV/chitosan composites show that in the comparison to the PHBV reference (*Xc* = 65%), the introduction of 10 wt.% chitosan did not change the crystallinity significantly. In functionalized composites, the degree of crystallinity increases with an increase in the mass fraction of chitosan. In the case of composites functionalized with GMA, the degree of crystallinity is even higher than in the PHBV reference, up to more than 80%.

Possible factors that might play a role in increasing the crystallinity are the effect of chitosan as a nucleating agent due to a strong interfacial reaction between chitosan and the GMA-functionalized polymer chains. A similar observation was made by Li et al. [33]. Their article describes an increase of the enthalpy of fusion and thus of the degree of crystallization by interfacial reactions between GMA-grafted PHBV chains with carboxylic acid terminated polypropylene carbonates. They also suggested that the PHBV-g-GMA acts as nucleating agent, facilitating the heterogeneous crystallization process.

In summary, chitosan has a small nucleating effect on PLA and thus contributes to the crystallization of the polymer. Furthermore, GMA functionalized PHBV in combination with chitosan show a strong increase in crystallinity, which indicates an interfacial reaction between the two species. This nucleating effect, together with the shift in *Tg* values for the composite, are indicators of an enhanced compatibility of the phases.

#### 3.2.5. FTIR Characterization

When a radical-initiated grafting of maleic anhydride takes place, the transformation into a saturated agent, i.e., succinic anhydride, also happens (see Figure 1). Pure succinic anhydride shows characteristic absorption bands in the FTIR spectogram for the symmetrical stretching oscillation of the carbonyl group of the cyclic anhydride at 1775 cm^−1^ (strong) and for the asymmetrical stretching oscillation of the carbonyl group at 1859 cm^−1^ (weak). Grafted onto PLA or PHBV, the molecular environment of the grafted agent changes so that there is a slight shift of these bands. According to the literature, the characteristic bands for the symmetric C=O in PLA-g-MAH are found at 1780 cm^−1^ [8,34] the asymmetric C=O in PLA-g-MAH and PHBV-g-MAH at 1849–1850 cm^−1^ [7,8,34]. The absorption band corresponding to the C-H bending of the anhydride ring has been reported at 695 cm^−1^ for PLA-g-MAH [9] and at 690 cm^−1^ for PHBV-g-MAH [14].

For the PLA-g-GMA, a clearly visible absorption band for the epoxy group of about 910 cm^−1^ should appear [27,35]. No FTIR spectrum analysis for PHBV-g-GMA was found.

There are no characteristic bands for the oscillations of the cyclic anhydride compounds in the spectra of the PLA-g-MAH samples. Detyothin et al. [10], who spectroscopically examined PLA grafted with MAH and could not detect any characteristic peaks, assumed that the carbonyl band of MAH was covered by the PLA carbonyl group, due to its low concentration. According to the results of this study, this assumption cannot be confirmed because there is no absorption of neat PLA in the range of 1850 cm^−1^. In PHBV-g-MAH polymers, this characteristic peak is also absent. Mani et al. [8] and Zhu et al. [34] only observed this peak using second derivative spectra for enhancing the resolution.

**Figure 9:**
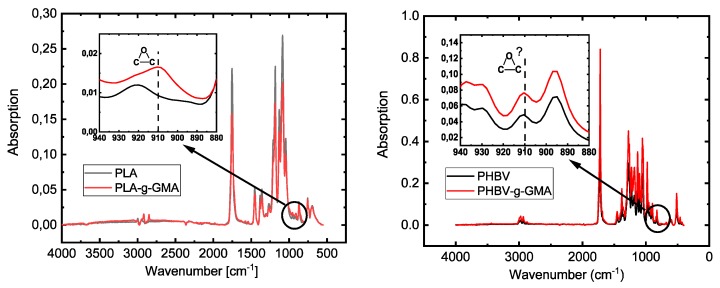
FTIR for GMA grafted PLA (**left**) and PHBV (**right**).

In contrast to the MAH grafted polymers, the GMA functionalized PLA samples show the epoxy band blends at 910cm^−1^ as seen in Figure 9, left. However, this band was not clear in the PHBV-g-GMA spectrum, probably because at this wavenumber, the neat PHBV also displays an absorption peak.

The measurement of the composites with chitosan revealed new absorption bands in the spectrogram, which could be associated with amide compounds. Secondary or tertiary amides were searched for in the spectral interpretation of the composites with MAH functionalized polymers, whereas in the composites functionalized with GMA, the search was limited to hydroxides and secondary amides. In general, it can be said that the ability of primary and secondary amides to form hydrogen bonds and the cis-trans isomerism of mono-substituted amides generally complicates the interpretation of the spectrum [36]. Table 5 shows the possible absorption bands that may appear in the composites prepared.

**Figure 10:**
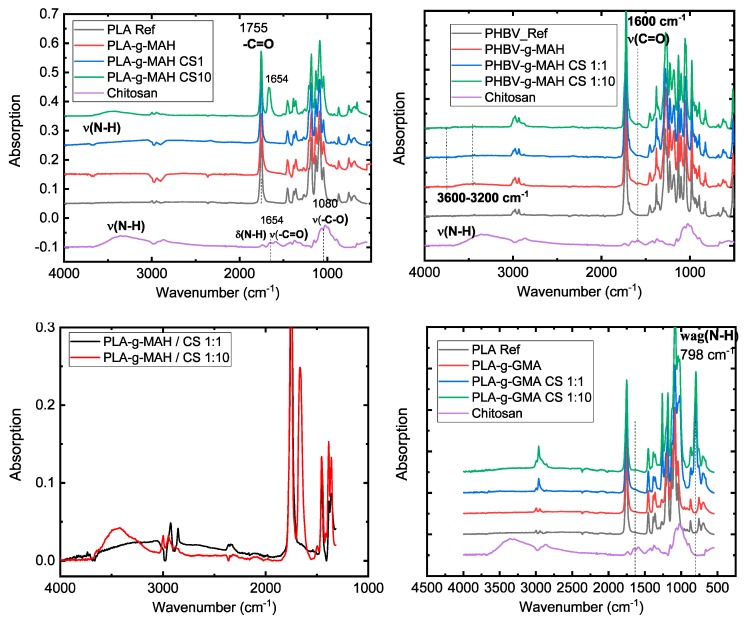
FTIR of the composites: Top-left (PLA-g-MAH) with chitosan; top-right (PHBV-g-MAH) with chitosan; bottom-left (PHBV-g-MAH) with chitosan zoom-in and bottom-right PLA-g-GMA.

For the secondary and tertiary amides, the carbonyl groups absorb between 1680 and 1630 cm^−1^. The secondary amides show an additional ν(N–H) control band at 3300 cm^−^^1^. In the PLA-g-MAH composites with chitosan, a vibration of carbonyl at 1640 cm^−^^1^ is clearly seen in the green spectra in Figure 10 (top-right) and a weak increase in the range from 3500 to 3000 cm^−^^1^ is recognized for the control band. At 1550 cm^−^^1^, there is a band which can be assigned to the interaction of the ν(C–N) valence oscillations with the δ(CNH) deformation vibration.

A characteristic δ(N–H) oscillation for secondary amides should be detected at 1250 cm^−1^, but this is not found in the spectra. For both polymers grafted with MAH, the spectra with different chitosan fractions are shown in Figure 10. The N–H control bands are only weakly pronounced in both polymers. However, characteristic amide bands for each polymer could only be determined for the highest chitosan portion. This is not in agreement with the titration analysis. Regarding the type of bonds, tertiary amides (imide compounds) seem to be the predominant bonds in the composite due to the weakly pronounced N–H oscillations.

For the composites functionalized with GMA, strong bands in the range of 3600 to 3100 cm^−1^ can be identified for both polymers. They can be assigned to hydroxide vibrations. Furthermore, in the case of PLA-g-GMA/Chitosan composites, an absorption band at 1550 cm^−1^ can be identified. This can be assigned to the interaction between the ν(C–N) valence oscillation of the δ(N–H) deformation vibration. New bands at 798 cm^−1^ appear in PLA-g-GMA composites only in the presence of chitosan, assumed for wag(N–H) in the secondary amines (see Figure 10 bottom-right.). In PHBV-g-GMA composites, a weak band at 1640 cm^−1^ indicates secondary amide bonds. It can be assumed that as shown in Figure 7, these bonds formed in the composites are amino alcohols.

The difference in the functional groups that can be identified for both grafted polymers with chitosan denotes that regardless of the similarity of the polyesters and the expected reactions, each functionalized polymer shows different structures.

#### 3.2.6. Scanning Electron Microscopy

Scanning electron microscopy (SEM) was used to observe the morphology of the various chitosan composites from the internal mixer. The functionalized polymers compounded with chitosan (PLA/PHBV_GMA/MAH_CS) and, as references, the non-functionalized polyesters processed with chitosan (PLA/PHBV_CS) were investigated. This method was chosen to check whether the chitosan particles were bonded to the functionalized polymer matrix. A good binding of particles in a composite can be recognized by the gap distance between particles and matrix. In case of successful compatibilization, the gap may completely disappear. The images show the top view of the fracture surface, formed after the cryogenic fracture of the specimens.

**Figure 11:**
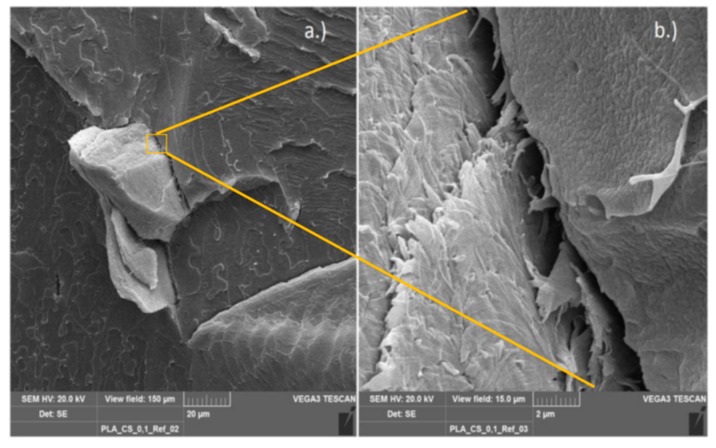
Micrograph, PLA reference with 10% chitosan.

The SEM image of the PLA/chitosan reference with a 10 wt.% chitosan content (see Figure 11) shows a chitosan particle protruding from the polymer matrix with a diameter of approx. 45–50 μm. An enlarged image clearly shows a gap between the particle and polymer matrix, indicating poor or no binding at all. A further indication is the integrity and the perpendicular protrusion of the particle. If it were well bonded to the matrix, it would rather break, caused by the force transmission in breaking the sample.

**Figure 12:**
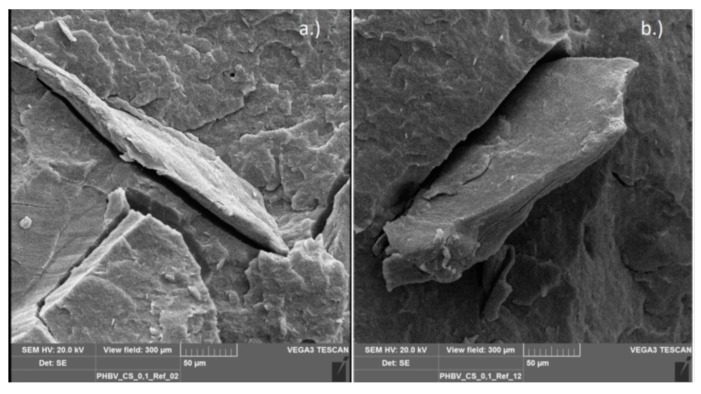
SEM-micrograph of PHBV reference with 10% chitosan.

Figure 12 shows the chitosan-PHBV reference, again containing 10 wt.% mass fraction of chitosan. The images are comparable to those of the PLA reference. In this particular image, the chitosan particles are larger and elongated, about 150 μm in size. The gaps between particle and matrix as well as the integrity of the particle are even more pronounced than for the PLA/chitosan compound.

**Figure 13:**
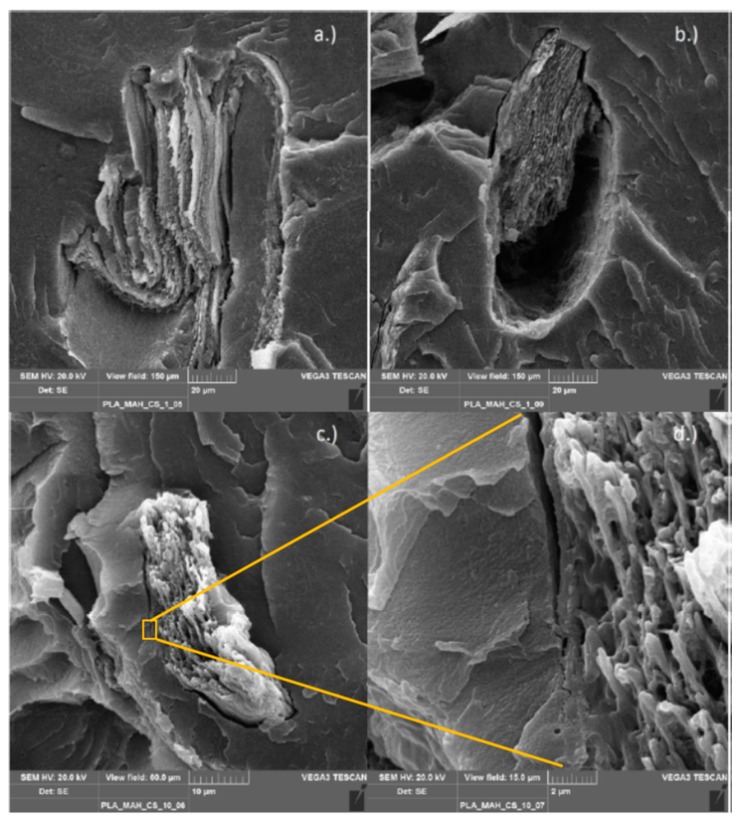
PLA-g-MAH compounded with 1% chitosan (**a**,**b**) and 10% chitosan (**c**,**d**).

Figure 13 shows a compilation of SEM images of the PLA/chitosan composites functionalized with MAH. Several 50–25 μm large chitosan particles embedded in the polymer matrix with partly good and partly bad binding can be observed. Most of the particles are broken in the middle, which can be seen from the different surface structures. For example, Figure 13a shows a strongly deformed chitosan particle broken in the middle. This particle breakage indicates good bonding to the PLA matrix and high shear force transfer during processing. Figure 13b shows a chitosan particle that seems to be poorly bonded because it was broken out on its right side. However, this fact exactly indicates that the particle had a very good binding to the polymer matrix, at least on the left side. Instead of breaking out completely, it was retained by the matrix and cracked. Figure 13c shows another smaller particle (about 25 μm), having a slit that narrows at magnification Figure 13d and finally, tapers. This example also indicates good compatibility between chitosan particles and polymer matrix induced by MAH grafting.

**Figure 14:**
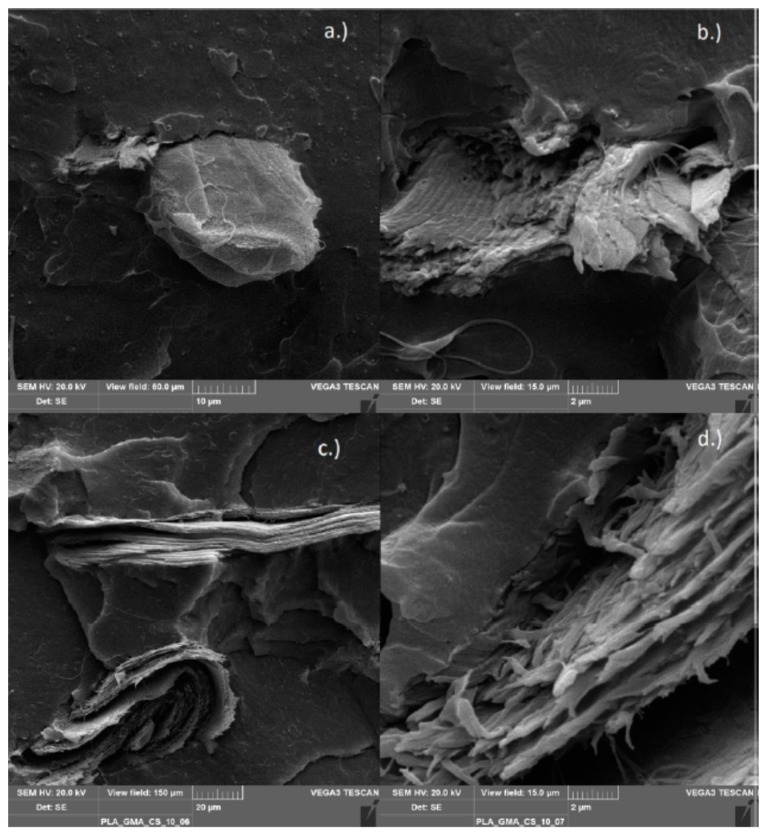
PLA-g-GMA compounded with 1% chitosan (**a**,**b**) and 10% chitosan (**c**,**d**).

Figure 14 shows an SEM image of the PLA-g-GMA composites with chitosan. In (a) an approx. 20 μm wide ellipsoid chitosan particle is visible, with an adjacent smaller one which is broken. While the intact chitosan particle shows non-optimal binding due to open gaps, a partially gapless transition of the smaller particle to the polymer matrix can be seen in the close-up image in (b). In (c) two broken chitosan particles are shown, one rod-shaped and the other strongly deformed, both showing a very good binding, as the close-up in (d) reveals.

**Figure 15:**
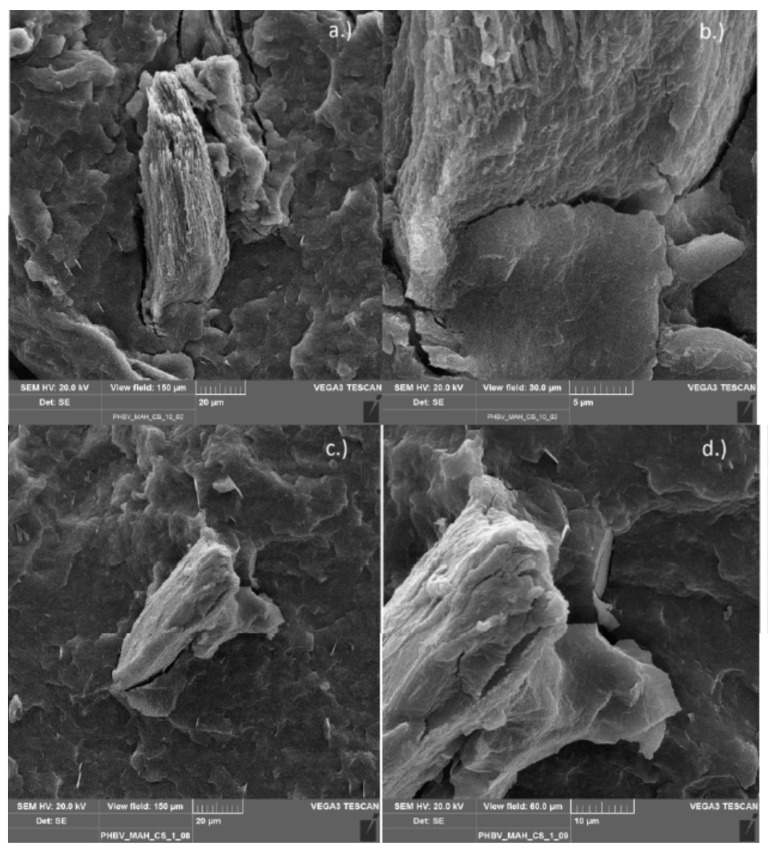
PHBV-g-MAH compounded with 10% chitosan (**a**,**b**) and with 1% chitosan (**c**,**d**).

Figure 15 shows the PHBV/chitosan composite functionalized with MAH, in image (a) and (c) two different particles are presented, each with a magnification in images (b) and (d). It is evident that there are several tapering gaps (see (b)), or even no gaps at all between particle surface and polymer (see d)).

**Figure 16:**
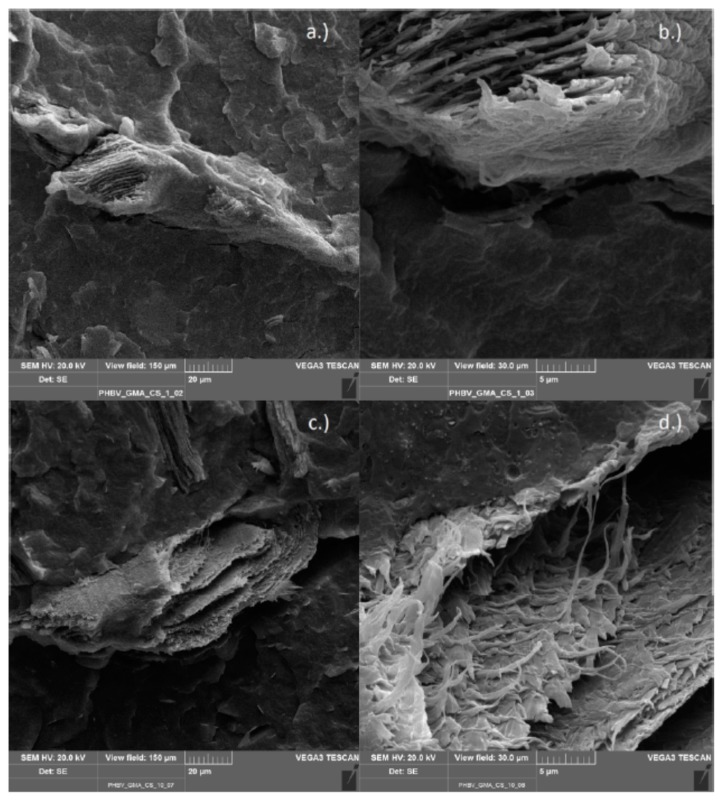
PHBV-g-GMA compounded with 10% chitosan (**a**,**b**) and with 1% chitosan (**c**,**d**).

The PHBV/chitosan composite functionalized with GMA is shown in Figure 16. In (a) and (b) a chitosan particle is shown, which is apparently well coated with the polymer matrix and which can only be identified as chitosan particle due to its internal structure. In (c) and (d) a larger and broken particle is shown having a seamless transition to the polymer matrix. On closer inspection, in (d) smaller fragments of chitosan particles are also visible, which probably were distributed in the matrix during processing.

In summary, a lot of differences between the references and the compatible chitosan composites can be detected under the scanning electron microscope. While in the reference materials, only unbroken particles with comparatively wide gaps between matrix and particles are recognizable, the chitosan particles in the composites show fractures and very small or even scarcely existing gaps. These aspects hint to partially good binding of the particles to the matrix caused by the functionalization of the polymers.

#### 3.2.7. Mechanical Properties

Another possibility for evaluating successful binding of the chitosan particles to the polymer matrix is the investigation of the mechanical properties of the compounds. The values determined by tensile tests, such as modulus of elasticity, tensile strength and breaking stress allow deducing as to whether the material properties, such as strength or brittleness, become improved or deteriorated in conjunction with chitosan. In general, an improvement of the mechanical properties would therefore mean a good bonding of the chitosan in the composite.

**Figure 17:**
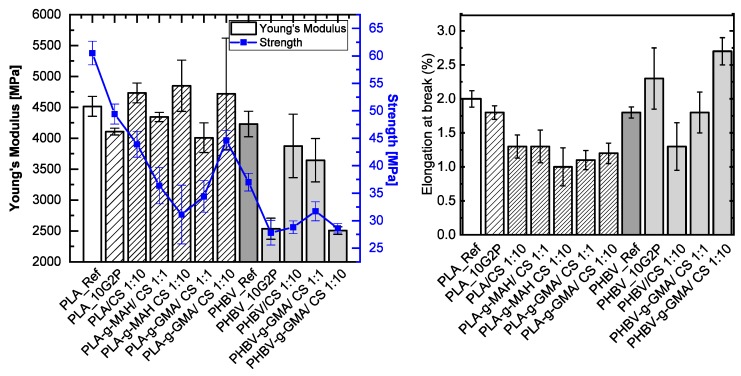
Tensile properties of the composites. **Left**: Young’s Modulus and tensile strength. **Right**: Elongation at break.

Some tensile samples could not be prepared due to the brittleness of the material. That was the case for PLA-g-MAH and PHBV-g-MAH. The composites of PHBV-g-MAH with chitosan could also not be prepared; however, the composites of PLA-g-MAH with chitosan could be measured. The brittleness is related to the lower molecular weight of the samples, as indicated previously. Figure 17 summarizes the results of the tensile tests. For PLA samples, the Young’s modulus shows no trend and high error bars. The strength decreases in all the PLA samples in comparison with the reference PLA sample. This is caused either by the thermal degradation during processing or by the strength of the chitosan particles. The PLA-g-MAH samples show a significant decrease in strength. Only the sample PLA-g-GMA with 1:10 Chitosan shows a slightly higher strength. The elongation at break was also reduced with the addition of chitosan in PLA samples. On the other hand, the samples of PHBV show a different behavior. An increase in the Young’s modulus with the addition of chitosan is observed in relation with the reference sample PHBV-g-GMA (PHBV_10G_2P), although for the functionalized sample, with 1:10 chitosan, the trend is reversed. Another change to be highlighted is the increase in elongation at break with PHBV/CS-compounds and GMA grafting, considering that during processing a small decrease in molecular weight was observed and no significant change in crystallinity was measured. With the functionalization of the polymer chains with GMA, the compatibility of PHBV with chitosan increases, leading to a higher elongation capacity. These results may corroborate the good adhesion of the chitosan particles, especially for the GMA functionalized polymers, as already seen in the SEM micrograph.

## 4. Conclusions

This work presents a practical approach for the functionalization of thermoplastic biodegradable polyesters using reactive extrusion for enhancing the compatibility with amine-functionalized particles or polymers. A single step process without any previous treatment of the raw materials was performed for producing biodegradable composites with chitosan fillers, which can be extrapolated to conventional extrusion compounding.

The functionalization of the polymers with MAH and GMA in the melt state can be achieved by using radical initiators in a reactive extrusion. However, the yields for these reactions are low and the control for avoiding side reaction is complex. Titration methods can be used for the quantification of functional groups covalently attached to the polymer, although it is not possible to determine whether the groups are randomly distributed in the polymer chains. The success of grafting can partially be confirmed with FTIR, but the grafting degree is too low to be quantified using this analytical method.

The peroxide reaction proceeds quickly, increasing the viscosity. However, thermal degradation of the polymers simultaneously takes place, leading to lower molecular weights. The increase in viscosity is a clear indicator of chain extension or branching reactions on the polymer matrix. From the difference between the molecular weight achieved with MAH and GMA, it can be inferred that additional reaction of the epoxy groups with terminal acids may occur compensating the decrease in molecular weight. Additional kinetic studies are needed to ensure the proper balance between branching, chain extension and thermal degradation.

The composites with chitosan did not show any particular enhancement in thermal or tensile properties. The functionalization of PHBV with GMA decreases the glass transition temperature of the polymer, while MAH has no significant influence, probably because of the smaller size of the functional group. Nevertheless, the SEM pictures clearly show an enhanced compatibility, especially for GMA grafted polymers with chitosan.

When analyzing all of the results obtained through the different techniques used in this work together, it is possible to conclude that the functionalization with GMA effectively increases the compatibility of the polyester matrix with the chitosan particles, obtaining composites where the mechanical properties are not severely affected.

Moreover, these results can be considered as a proof of concept for preparing polyester compounds with chitosan in a single-step process through reactive extrusion.

Future works should be focused on correlating the influence of the residence time in the extrusion process to avoid hydrolysis and thermal degradation and to ensure a complete reaction of the grafted polymer with the functional filler. The kinetic of peroxide reaction with polyesters is fast in relation with other reactions and can easily be performed in extrusion processes with short residence times.

## Figures and Tables

**Table 1:** Samples prepared for grafting and codification.

Sample Name	Polymer	Grafting Agent	wt.% of Grafting Agent	wt.% of Peroxide
PLA_Ref.	PLA	None	0%	0%
PLA Ref. PHX	PLA	None	0%	0.30%
PLA3M1P	PLA	MAH	3%	0.30%
PLA10M1P	PLA	MAH	10%	0.30%
PLA3M2P	PLA	MAH	3%	0.60%
PLA10M2P	PLA	MAH	10%	0.60%
PLA3G1P	PLA	GMA	3%	0.30%
PLA10G1P	PLA	GMA	10%	0.30%
PLA3G2P	PLA	GMA	3%	0.60%
PLA10G2P	PLA	GMA	10%	0.60%
PHBV_Ref.	PHBV	None	0%	0%
PHBV_Ref. PHX	PHBV	None	0%	0.30%
PHBV_3M1P	PHBV	MAH	3%	0.30%
PHBV_10M1P	PHBV	MAH	10%	0.30%
PHBV_3M2P	PHBV	MAH	3%	0.60%
PHBV_10M2P	PHBV	MAH	10%	0.60%
PHBV_3G1P	PHBV	GMA	3%	0.30%
PHBV_10G1P	PHBV	GMA	10%	0.30%
PHBV_3G2P	PHBV	GMA	3%	0.60%
PHBV_10G2P	PHBV	GMA	10%	0.60%

**Table 2:** Sample preparation and codification for composites with chitosan.

Sample Name	% Peroxide	% Chitosan	nNH2:nMAH or nNH2:nGMA
PLA-g-MAH_CS 1:10	0.6	12.4%	1:10
PLA-g-MAH/Chitosan 1:1	0.6	1.2%	1:1
PHBV-g-MAH/Chitosan 1:10	0.3	20.1%	1:10
PHBV-g-MAH/Chitosan 1:1	0.3	2.0%	1:1
PLA-g-GMA/Chitosan 1:10	0.6	21.7%	1:10
PLA-g-GMA/Chitosan 1:1	0.6	2.2%	1:1
PHBV-g-GMA/Chitosan 1:10	0.3	44.8%	1:10
PHBV-g-GMA/Chitosan 1:1	0.3	4.5%	1:1

**Table 3:** Reaction of MAH grafted polymers with chitosan in the compound.

Sample Name	MAH in Composite	MAH without Chitosan	Reacted MAH
PLA-g-MAH/Chitosan 1:10	0.51%	5.70%	91%
PLA-g-MAH/Chitosan 1:1	0.47%	6.50%	93%
PHBV-g-MAH/Chitosan 1:10	0.56%	6.30%	91%
PHBV-g-MAH/Chitosan 1:1	0.56%	6.50%	91%

**Table 4:** Thermal properties of the polymers and compounds from DSC.

Probename	*Tg* * [°C]	*Tm* [°C]	*Xc*
PLA_Ref.	62.1	151.1	0.4%
PLA_PHX_Ref.	63.3	150.6	28.6%
PLA_3M1P	56.7	152.9	−0.4%
PLA_10M1P	57.5	148.3	0.5%
PLA_3M2P	59.5	152.8	1.8%
PLA_10M2P	46.3	149.4	2.3%
PLA_3G1P	55.5	147.4	24.0%
PLA_10G1P	61	148.9	−0.4%
PLA_3G2P	62.5	148.9	10.1%
PLA_10G2P	60.5	148.6	−0.4%
PHBV_Ref.	6.2	173.6	66.5%
PHBV_PHX_Ref.	8.2	168	61.0%
PHBV_3M1P	8.9	166.5	60.5%
PHBV_10M1P	7.5	168.5	60.7%
PHBV_3M2P	7.4	165.8	63.9%
PHBV_10M2P	9.1	162.8	57.0%
PHBV_3G1P	4.6	171.5	55.4%
PHBV_10G1P	3.7	171.4	57.9%
PHBV_3G2P	4.6	170.1	61.4%
PHBV_10G2P	4.3	168.1	68.8%
PLA/Chitosan (Ref.) 1:10	58.8	151.7	4%
PLA-g-MAH/Chitosan 1:10	59.5	149.6	0%
PLA-g-MAH/Chitosan 1:1	59.7	149.6	0%
PLA-g-GMA/Chitosan 1:10	60.1	148.6	0%
PLA-g-GMA/Chitosan 1:1	57.8	148.5	0%
PHBV/Chitosan (Ref.) 1:10	-	175.6	65%
PHBV-g-MAH/Chitosan 1:10	4.5	164.4	65%
PHBV-g-MAH/Chitosan 1:1	8.1	166.2	60%
PHBV-g-GMA/Chitosan 1:10	6.8	168.5	83%
PHBV-g-GMA/Chitosan 1:1	5	170.1	61%

* *Tg* determined from the inflection point.

**Table 5:** Expected absorption band for the composites.

Sample	Wavenumber Range [cm^−1^]	Vibration	Functional Group
PLA-g-MAH/CS	3500–3100	ν(N–H)	Secondary amide
	1640	ν(C=O)	Secondary or tertiary amide
	1550	ν(C–N) + δ(N–H)	Secondary amide
PHBV-g-MAH/CS	3600–3200	ν(N–H)	Secondary amide
	1600	ν(C=O)	Secondary or tertiary amide
PLA-g-GMA/CS	3600–2500	ν(O–H)	Hydroxide stretching from carboxilic acid
	1550 796	ν(C–N) + δ(N–H) wag (N–H)	Secondary amine Primary and secondary amine
PHBV-g-GMA/CS	3600–3100	ν(O–H)	Hydroxide
	1640	ν(C=O)	Secondary amide
